# Identification of Regions Critical for the Integrity of the TSC1-TSC2-TBC1D7 Complex

**DOI:** 10.1371/journal.pone.0093940

**Published:** 2014-04-08

**Authors:** Arthur Jorge Santiago Lima, Marianne Hoogeveen-Westerveld, Akio Nakashima, Anneke Maat-Kievit, Ans van den Ouweland, Dicky Halley, Ushio Kikkawa, Mark Nellist

**Affiliations:** 1 Department of Clinical Genetics, Erasmus Medical Center, Rotterdam, The Netherlands; 2 Biosignal Research Center, Kobe University, Kobe, Japan; National Cancer Institute, United States of America

## Abstract

The TSC1-TSC2-TBC1D7 complex is an important negative regulator of the mechanistic target of rapamycin complex 1 that controls cell growth in response to environmental cues. Inactivating *TSC1* and *TSC2* mutations cause tuberous sclerosis complex (TSC), an autosomal dominant disorder characterised by the occurrence of benign tumours in various organs and tissues, notably the brain, skin and kidneys. *TBC1D7* mutations have not been reported in TSC patients but homozygous inactivation of *TBC1D7* causes megaencephaly and intellectual disability. Here, using an exon-specific deletion strategy, we demonstrate that some regions of TSC1 are not necessary for the core function of the TSC1-TSC2 complex. Furthermore, we show that the TBC1D7 binding site is encoded by *TSC1* exon 22 and identify amino acid residues involved in the TSC1-TBC1D7 interaction.

## Introduction

*TSC1* and *TSC2* are tumour suppressor genes that are mutated in individuals with tuberous sclerosis complex (TSC) [1], [2]. TSC is a hamartoma syndrome characterised by the occurrence of benign tumour-like lesions in many different organs and tissues, including the brain, skin, kidneys, lungs and heart. Many individuals with TSC have epilepsy and cognitive and behavioral deficits [3].

*TSC1* and *TSC2* encode the TSC1 (130 kDa) and TSC2 (200 kDa) proteins. The C-terminal domain of TSC2 contains a small region of homology with GTPase-activating proteins (GAPs), and TSC1 and TSC2 interact to form a complex that acts as a GAP for the small G-protein RHEB, accelerating the conversion of RHEB from its active GTP-bound state to its inactive GDP-bound form [4]. RHEB-GTP is required for activation of the mechanistic target of rapamycin (mTOR) complex 1 (mTORC1) that stimulates cell growth by promoting protein translation and lipid synthesis, and inhibiting autophagy [5]. In low energy, low glucose conditions, the TSC1-TSC2 complex is activated to down-regulate TORC1 activity, whereas in response to growth factors, the TSC1-TSC2 complex is inactivated to allow RHEB-GTP-dependent stimulation of TORC1.

TSC1 and TSC2 are both required for full TSC1-TSC2 activity and whereas it is clear why the catalytic TSC2 subunit is essential, the exact role of TSC1 is less well defined. TSC1 stabilises TSC2 and prevents TSC2 ubiquitination and proteosomal degradation [6], helps maintain the TSC1-TSC2 complex in the correct intracellular localisation [7], [8] and regulates TSC1-TSC2 activity through diverse signalling pathways [9]. TSC1 and TSC2 form a stable complex due to interactions between the N-terminal domain (NTD) of TSC2 (amino acids 1 - 900) [10] and multiple regions in TSC1 [11], including a large predicted coiled coil close to the TSC1 C-terminus (amino acids 726 – 988) [2]. All confirmed pathogenic *TSC1* missense mutations identified to date destabilise TSC1 and map inside the hydrophobic core of the TSC1 NTD [12], between TSC1 amino acids 50 and 224 [13], [14].

Consistent with an important role for TSC1 in the regulation of TSC1-TSC2 activity, many interactions between TSC1 and other proteins have been identified [15]. Although the exact nature and importance of many of these interactions to TSC1-TSC2 function remains unclear [4], there is strong evidence for binding between TSC1 and the Tre2-Bub2-Cdc16 (TBC) 1 domain family, member 7 (TBC1D7) [16]–[18]. The TSC1-TSC2-TBC1D7 interaction helps stabilise these three proteins in the "Rhebulator" complex [17], [18].

TBC proteins act as GAPs for the RAB family of GTPases that play a major role in organelle trafficking and biogenesis [19]. Although TBC1D7 lacks both of the residues that are thought to be essential for catalytic GAP activity [19] it has been shown to specifically inactivate RAB17 and inhibit primary cilium formation [20]. Furthermore, both TBC1D7 over-expression [16] and RNAi-mediated *TBC1D7* knock-down [18] increased TORC1 activity. Although no *TBC1D7* mutations have yet been found in TSC patients [18], homozygous loss of *TBC1D7* causes intellectual disability, megaencephaly and increased TORC1 signalling [21]. In addition, brain tissue expression quantitative trait locus analysis identified *TBC1D7* as a susceptibility locus for migraine [22]. Here we use a targeted mutagenesis approach to investigate structure-function relationships in TSC1, and to further characterise the TBC1D7-TSC1 interaction.

## Materials and Methods

### TBC1D7 mutation analysis

Genomic DNA of 19 individuals with TSC in whom no *TSC1* or *TSC2* mutation had been identified was prepared using standard methods and the individual coding exons of *TBC1D7* were amplified by the polymerase chain reaction (PCR) using primers, as described previously [18]. PCR products were sequenced on an ABI3130 (Applied Biosystems, Foster City, U.S.A.). The study was approved by the Medical Ethics Committee of the Erasmus Medical Center and written informed consent was obtained from the relevant individuals (signed consent form).

### Constructs and antisera

Mammalian expression constructs were derived using the QuikChange site-directed mutagenesis kit (Stratagene, La Jolla, U.S.A.), as described previously [13]. Primer sequences used for site-directed mutagenesis are shown in Supporting lnformation Tables S1 and S2. Other mammalian expression constructs used in this study have been described previously [14], [16], [23] or were purchased from Addgene (pcDNA3-HA-H-RAS)(Cambridge, U.S.A.) or Invitrogen (pcDNA3-β-lactamase-myc)(Carlsbad, U.S.A.). Bacterial expression constructs were derived by PCR amplification from the corresponding mammalian expression construct or from cDNA prepared from human fibroblast RNA, followed by cloning into the pGEX-2T vector (Clontech, Mountain View, U.S.A.). In each case the complete open reading frame of the construct was verified by sequence analysis. DNA was prepared using the Plasmid Plus Midi Purification kit (Qiagen, Venlo, The Netherlands).

Antibodies were purchased from Cell Signaling Technology (Danvers, U.S.A.)(1A5, anti-T389 phospho-S6K mouse monoclonal; anti-myc tag rabbit polyclonal; 9B11 anti-myc mouse monoclonal; anti-HA tag rabbit polyclonal), Covance (Princeton, U.S.A.)(Mouse anti-HA tag) or Li-Cor Biosciences (Lincoln, U.S.A.) (goat anti-rabbit 680 nm and goat anti-mouse 800 nm conjugates. The rabbit polyclonal against glutathione-S-transferase (GST) was kindly provided by A. Hoogeveen and L. van Unen (Erasmus Medical Center, The Netherlands). Rabbit polyclonal antibodies raised against human TSC2 have been described previously [24].

### Transfection experiments

HEK 293T cells were grown overnight in full medium (Dulbecco's modified Eagle medium (DMEM) (Lonza, Verviers, Belgium) supplemented with 10% foetal bovine serum, 50 U/ml penicillin and 50 μg/ml streptomycin) in a 10% carbon dioxide humidified incubator at 37°C. Cells at 80 – 90% confluency were transfected using polyethyleneimine (PEI). In each experiment the expression constructs and PEI (1∶3 ratio) were incubated for 15 minutes prior to adding to the cells. After 4 hours the transfection mixtures were replaced with full medium. Twenty-four hours after transfection the cells were transferred to ice, washed with cold phosphate-buffered saline (PBS) and harvested in lysis buffer (50 mM Tris-HCl pH 8.0, 150 mM NaCl, 50 mM NaF and 1% Triton X100, containing a protease inhibitor cocktail (Complete, Roche Molecular Biochemicals, Woerden, The Netherlands)). After centrifugation (10 000 g for 10 minutes at 4°C), the supernatant fractions were recovered, diluted in loading buffer and incubated at 96°C for 5 minutes prior to electrophoresis on Criterion 4–12% SDS-PAGE gradient gels (Bio-Rad, Hercules, U.S.A.). Proteins were transferred to nitrocellulose membranes according to the manufacturer's recommendations. Blots were blocked with 5% low-fat milk powder (Campina Melkunie, Eindhoven, The Netherlands) in PBS prior to incubation with the appropriate antibodies diluted in PBS containing 0.1% Tween 20 (PBST) (Sigma-Aldrich Fine Chemicals, Poole, U.K.). After washing 3 times for 5 minutes in PBST, the blots were incubated for 1 hour at room temperature in the dark in PBST containing 1/10 000 dilutions of goat anti-rabbit 680 nm and goat anti-mouse 800 nm secondary antibodies. After washing 3 times for 5 minutes in PBST and once in PBS, the blots were scanned using the Odyssey Infrared Imager (Li-Cor Biosciences) at default intensity, medium quality, 169 μm resolution with 0 mm focus offset. The integrated intensities of the protein bands were determined using the Odyssey default settings with the 3 pixel width border mean average background correction method. The transfection-based immunoblot assay for assessment of TSC1-TSC2 activity has been described previously [13], [14].

### Coimmunoprecipitation experiments

HEK 293T cells were transfected and harvested as described above. TBC1D7-TSC1-TSC2 complexes were immunoprecipitated by gentle mixing with anti-myc or anti-HA affinity resins (Sigma-Aldrich) for at least 2 hours at 4°C. After washing 3 times with >20 volumes lysis buffer, the bound proteins were analysed by immunoblotting.

### GST pull-down experiments

*E. coli* BL21 bacteria were transformed with constructs encoding GST fusion proteins. Single colonies were grown at 37°C overnight in 2 ml LB containing 100 μg/ml ampicillin and then transferred to 50 ml prewarmed medium and grown at 30°C until an optical density (600 nm) of 0.6. GST fusion protein expression was induced with 1 mM IPTG for 3 hours at 30°C. Bacteria were collected by centrifugation (10 000 g, 15 minutes, 4°C), resuspended in 2 ml resuspension buffer (50 mM Tris HCl pH 8.0, 150 mM NaCl, 50 mM NaF, Complete protease inhibitor cocktail) and lysed by sonication, followeed by the addition of 1% Triton X100. The lysate was cleared by centrifugation (10 000 g, 15 minutes, 4°C) and bound to glutathione-sepharose beads (Roche Molecular Biochemicals) by gentle agitation overnight at 4°C, followed by extensive washes with resuspension buffer containing 1% Triton X100. Yields and purity of the GST-fusion proteins were checked on coomassie-stained SDS-PAGE gels.

### In silico analyis

Protein sequence alignments were performed in DNAMan (Lynnon Corporation, Quebec, Canada). Coiled coil predictions were performed using the COILS server (http://www.ch.embnet.org/software/COILS_form.html) and analysis of potential phosphorylation sites was carried out using Scansite (http://scansite3.mit.edu/#home). Structural modelling was performed with the PyMol software package version 1.6 (Schrodinger, Mannheim, Germany).

## Results

### Deletion of different TSC1 coding exons have distinct effects on TSC1 function

**Figure 1:**
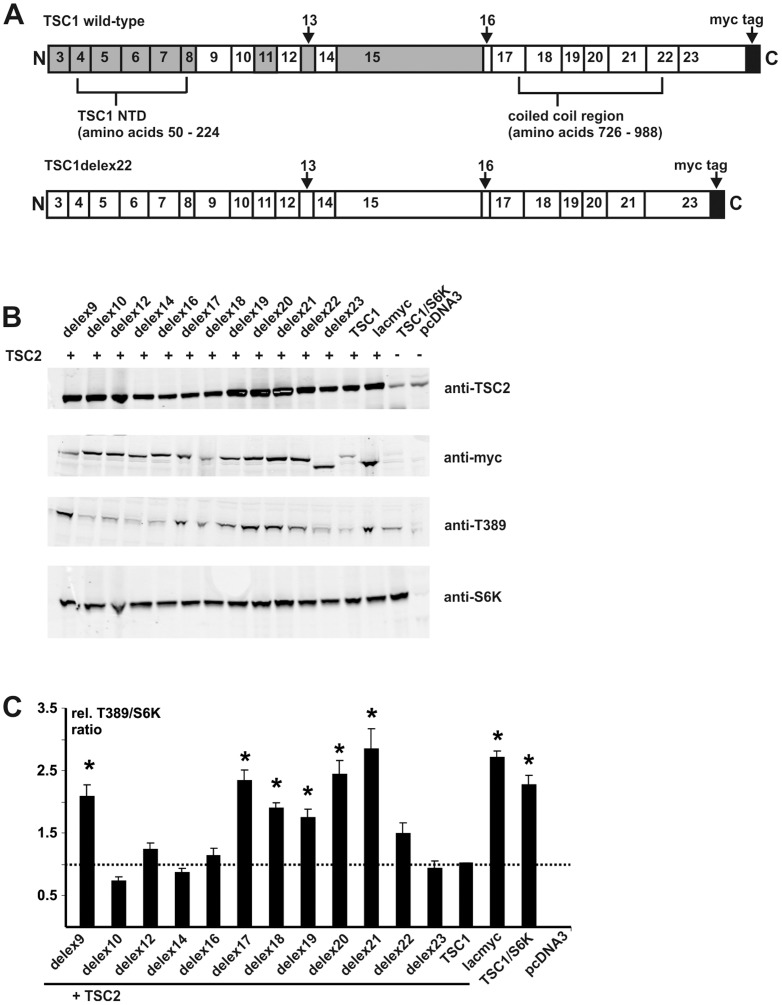
Functional assessment of TSC1 exon-specific deletions. Sequences corresponding to the individual coding exons of *TSC1* were deleted from a wild-type TSC1 expression construct so as to maintain the original reading frame (see Supporting Information Table S1). Each expressed TSC1 protein carried a C-terminal myc epitope tag to allow specific and unbiased detection. Each TSC1 exon-specific deletion protein (TSC1delex) was coexpressed with TSC2 and S6K and the TSC2, TSC1, S6K and T389-phosphorylated S6K signals were estimated by immunoblotting. Transfected HEK 293T cells expressing the TSC1delex proteins were compared to cells expressing wild-type TSC1. Cells expressing myc-tagged B-lactamase (lacmyc), S6K and TSC2, cells expressing wild-type TSC1 and S6K only (TSC1/S6K), and mock transfected cells (pcDNA3) were included as controls. A. Schematic overview of TSC1 (top) and TSC1delex22 (bottom). The segments encoded by each *TSC1* coding exon and the extent of the N-terminal domain (NTD) and coiled coil region are indicated. The position of the myc epitope tag is indicated by the black segment. No TSC1delex construct was derived for the exons shaded in grey. Note that TSC1delex22 lacks the segment corresponding to exon 22. B. Immunoblot analysis of the TSC1delex variants. Signals for TSC2 (anti-TSC2), the TSC1delex proteins (anti-myc), T389-phosphorylated S6K (anti-T389) and total S6K (anti-S6K) are shown. C. The integrated intensities of the T389-phosphorylated S6K (T389) and total S6K (S6K) immunoblot signals were quantified and the T389/S6K ratio was calculated relative to wild-type TSC1-TSC2 (wild-type TSC1 T389/S6K ratio = 1) in at least 3 independent experiments. TSC1delex variants with a significantly increased T389/S6K ratio (delex9, delex17, delex18, delex19, delex20 and delex21) (unpaired t-test p values<0.05) are indicated with asterisks. Error bars indicate the standard error of the mean.

To obtain insight into the domain structure of TSC1, we generated expression constructs lacking the amino acids encoded by exons 9, 10, 12, 14, 16, 17, 18, 19, 20, 21, 22 and 23 of *TSC1* (referred to as TSC1delex9, TSC1delex10 and so on; Figure 1A and Supporting Information Table S1) and expressed the resulting TSC1 in-frame deletion variant proteins in HEK 293T cells. In each case the expressed protein carried a C-terminal myc epitope tag. We were unable to derive expression constructs lacking the sequences encoded by exons 11, 13 or 15 due to difficulties designing appropriate site-directed mutagenesis primers. We did not assess exons 1 – 8. Exons 1 and 2 are non-coding, and exons 3 – 8 encode the TSC1 NTD that is critical for TSC1 stability [11], [12], [25].

First, we compared the effects of the different deletions on the TSC1-TSC2-dependent inhibition of TORC1 signalling by assessing the T389 phosphorylation status of S6K, as described previously [13](Figure 1B and C). Deletion of amino acids 247 – 304 (exon 9; TSC1delex9), 682 – 736 (exon 17; TSC1delex17), 737 – 797 (delex18), 798 – 834 (delex19), 835 – 875 (delex20) or 876 – 938 (delex21) significantly reduced the TSC1-TSC2-dependent inhibition of S6K-T389 phosphorylation, relative to full-length TSC1. In contrast, deletion of amino acids 306 – 343 (delex10), 382 – 421 (delex12), 445 – 479 (delex14), 667 – 680 (delex16) or 993 – 1164 (delex23) did not significantly affect the ability of TSC1-TSC2 to inhibit S6K-T389 phosphorylation. Deletion of amino acids 938 – 991 (delex22) resulted in a slight, but not significant, increase in S6K-T389 phosphorylation (Figure 1A and C).

**Figure 2:**
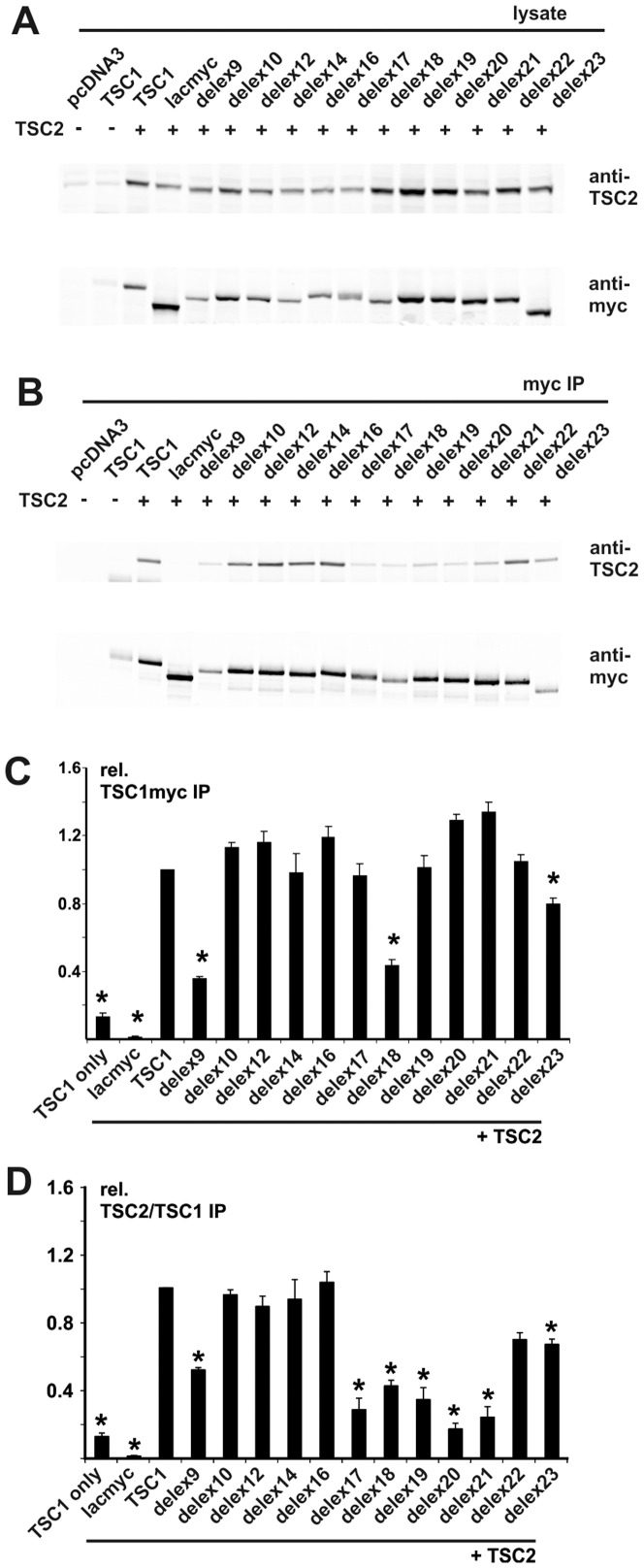
Interactions between TSC2 and the TSC1delex proteins. C-terminal myc-tagged TSC1delex proteins were co-expressed with TSC2 in transfected HEK 293T cells. TSC1-TSC2 complexes were immunoprecipitated with an antibody specific for the myc tag. The expressed proteins and immunoprecipitated complexes were analysed by immunoblotting. Cells expressing the TSC1delex proteins were compared to cells expressing TSC2 and wild-type TSC1, cells expressing TSC2 and myc-tagged β-lactamase (lacmyc), cells expressing wild-type TSC1 only, and mock transfected cells (pcDNA3). A. Immunoblot showing expression of TSC2 and the TSC1delex variants in the transfected cells. Signals for TSC2 (anti-TSC2) and the TSC1delex proteins (anti-myc) are shown. B. Immunoblot of the immunoprecipitated TSC1delex variants (anti-myc) and coimmunoprecipitated TSC2 (anti-TSC2). Signals for TSC2 (anti-TSC2) and the TSC1delex proteins (anti-myc) are shown. C. The integrated intensities of the immunoblot signals for the immunoprecipitated TSC1delex proteins were quantified relative to wild-type TSC1 (TSC1 = 1) in at least 3 independent experiments. TSC1delex proteins delex9, delex18 and delex 23, showed a significant decrease in the amount of immunoprecipitated TSC1 (unpaired t-test p values<0.05; indicated with asterisks). Error bars indicate the standard error of the mean. D. The integrated intensities of the immunoblot signals for coimmunoprecipitated TSC2 were quantified relative to wild-type TSC1-TSC2 (TSC1 = 1) in at least 3 independent experiments. The ratio of the TSC2 and TSC1 signals were calculated to normalise for the amount of immunoprecipitated TSC1delex protein. TSC1delex proteins delex9, delex17, delex18, delex19, delex20, delex21 and delex 23, showed a significant decrease in the amount of coimmunoprecipitated TSC2 (unpaired t-test p values<0.05; indicated with asterisks). Error bars indicate the standard error of the mean.

To investigate the effects of the deletions on the TSC1-TSC2 interaction we immunoprecipitated the different TSC1 exon deletion proteins and assessed TSC2 coimmunoprecipitation by immunoblotting. As shown in Figure 2, deletion of the amino acids corresponding to exon 9, 18 or 23 significantly reduced the amount of immunoprecipitated TSC1 as well as the amount of coimmunoprecipitated TSC2, whereas deletion of the amino acids corresponding to exons 17, 19, 20 or 21 only reduced the amount of coimmunoprecipitated TSC2. Deletion of the amino acids corresponding to exons 10, 12, 14, 16 or 22 did not significantly affect TSC2 coimmunoprecipitation.

### Deletion of TSC1 amino acids 938 – 991 disrupts the TSC1-TBC1D7 interaction

**Figure 3:**
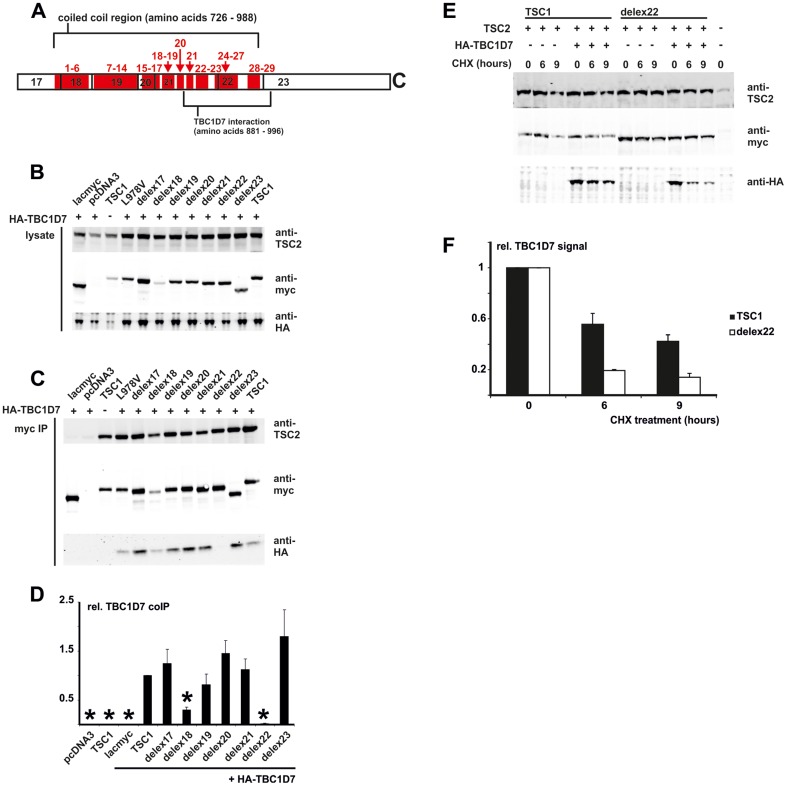
Interactions between TSC2, TBC1D7 and the TSC1delex proteins. TSC1delex proteins were co-expressed with TSC2 and HA-TBC1D7 in transfected HEK 293T cells, and TSC1-TSC2-TBC1D7 complexes were immunoprecipitated with an anti-myc tag antibody. Cells expressing the TSC1delex proteins were compared to cells expressing TSC2 and TBC1D7 only, TSC2 and TBC1D7 with myc-tagged wild-type TSC1 (TSC1), the TSC1 L978V variant or myc-tagged β-lactamase (lacmyc), and TSC2 and TSC1 only. A. Overview of the predicted TSC1 coiled coil region. Amino acids 681 – 1164, encoded by *TSC1* exons 17 – 23 is shown. The coiled coil region (amino acids 726 – 988) and the TBC1D7 binding domain (amino acids 881 – 996) are indicated. Heptad repeats 1 – 29 are indicated in red. B. Immunoblot showing the expression of TSC2, TBC1D7 and the TSC1delex variants. Signals for TSC2 (anti-TSC2), TBC1D7 (anti-HA) and the TSC1delex proteins (anti-myc) are shown. C. Immunoblot showing the immunoprecipitated TSC1delex variants and coimmunoprecipitated TSC2 and TBC1D7. Signals for TSC2 (anti-TSC2), TBC1D7 (anti-HA) and the TSC1delex proteins (anti-myc) are shown. D. The integrated intensities of the immunoblot signals for coimmunoprecipitated TBC1D7 were quantified relative to wild-type TSC1 (TSC1 = 1) in at least 3 independent experiments. TSC1 delex18 and delex22 showed a significant decrease in the amount of coimmunoprecipitated TBC1D7 (unpaired t-test p values<0.05; indicated with asterisks). Error bars indicate the standard error of the mean. E. Immunoblot of transfected HEK 293T cells expressing HA-TBC1D7, TSC2 and either wild-type TSC1 or TSC1delex22, and treated with cycloheximide (CHX) for 3, 6 or 9 hours. Signals for TSC2 (anti-TSC2), TBC1D7 (anti-HA) and TSC1/TSC1delex22 (anti-myc) are shown. F. The integrated intensities of the immunoblot signals for TBC1D7 in the CHX-treated cells were quantified relative to wild-type TSC1 (TSC1 = 1) in at least 3 independent experiments. TBC1D7 stability was decreased in the presence of TSC1delex22 compared to wild-type TSC1. Error bars indicate the standard error of the mean.

The TSC1 coiled coil region is predicted to consist of 29 complete heptad repeat sequences interspersed with short linker sequences (Figure 3A). Each heptad consists of hydrophilic residues in the *b*, *c*, *e*, *f* and *g* positions separated by hydrophobic residues at the *a* and *d* positions [25]. A previous study had shown that TBC1D7 bound to TSC1 amino acids 881 – 996 [16], corresponding to heptads 21 – 29. To investigate the effect of the *TSC1* exon deletions on the interaction with TBC1D7 we coexpressed TSC1delex17, TSC1delex18, TSC1delex19, TSC1delex20, TSC1delex21, TSC1delex22 and TSC1delex23 with TSC2 and TBC1D7 and assessed coimmunoprecipitation of TSC2 and TBC1D7 with the TSC1delex proteins by immunoblotting. TBC1D7 was coimmunoprecipitated together with all the TSC1delex proteins, except TSC1delex22 (Figure 3B – D), indicating that amino acids 938 – 992, corresponding to heptads 25 – 29 of the coiled coil domain, that are absent from TSC1delex22, are required for the TSC1-TBC1D7 interaction. In earlier work we had characterised a *TSC1* c.2932C>G (p.L978V) missense variant encoded by exon 22 [14]. This amino acid substitution did not prevent TBC1D7-TSC1 binding (Figure 3C). To confirm the importance of amino acids 938 – 991 for the TSC1-TBC1D7 interaction, we compared the effect of TSC1 and TSC1delex22 expression on TBC1D7 stability. Consistent with previous studies showing that the TBC1D7-TSC1 interaction is important for TBC1D7 stability [18], TBC1D7 was degraded more rapidly in cycloheximide-treated cells expressing TSC1delex22 than in cycloheximide-treated cells expressing wild-type TSC1 (Figure 3E and F).

### TSC1 amino acids 939 – 977 are sufficient for binding TBC1D7

**Figure 4:**
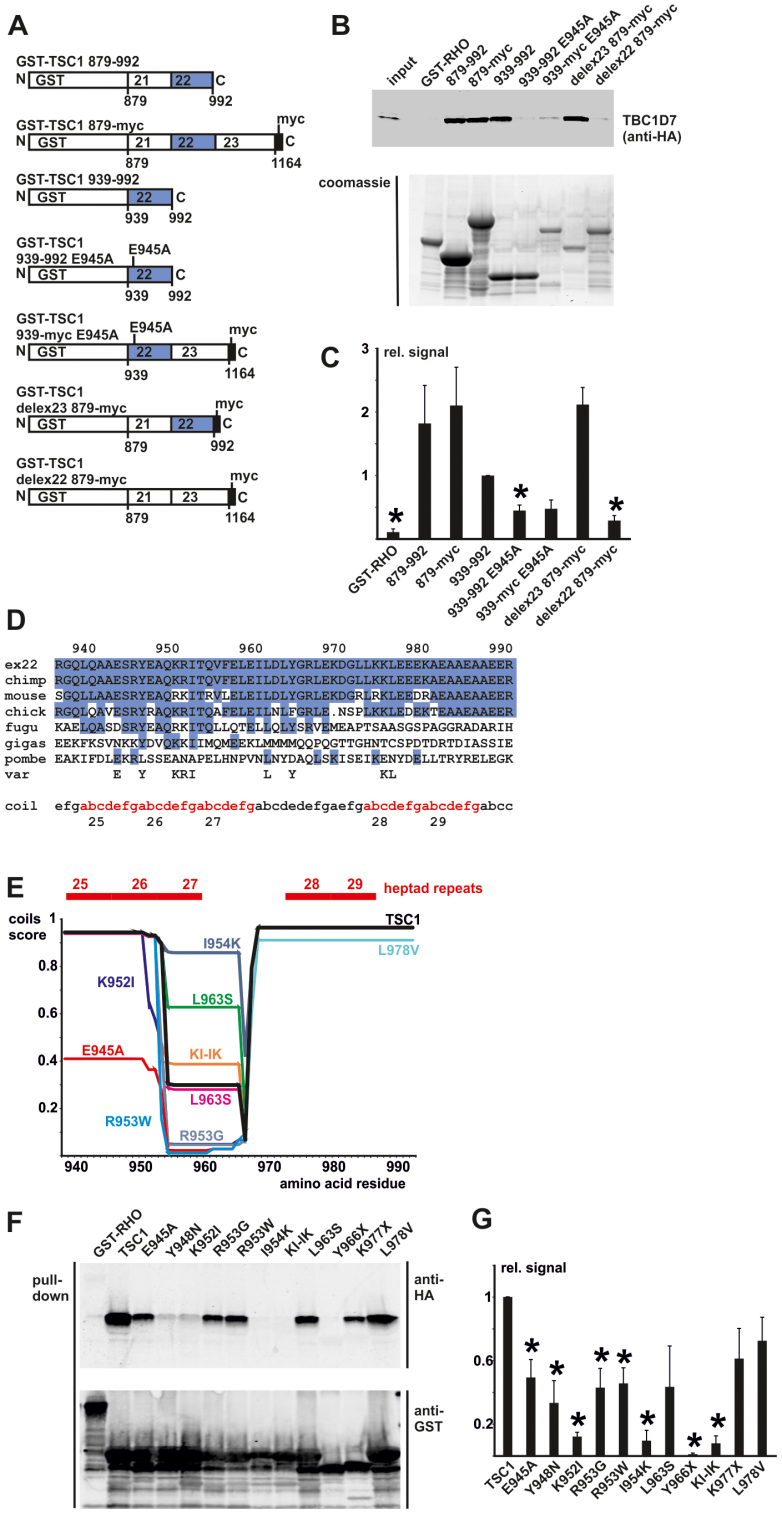
TSC1 amino acids 939 – 977 are sufficient for binding TBC1D7. HA-TBC1D7 was expressed in transfected HEK 293T cells. Glutathione-S-transferase (GST) TSC1 fusion proteins were expressed in *E. coli* and purified on glutathione-agarose beads. Interactions between TBC1D7 and the GST-TSC1 fusion proteins were assayed by glutathione bead pull-down followed by immunoblotting. A. Overview of the GST-TSC1 fusion proteins. Sequences corresponding to exon 22 are shaded; the E945A substitution and myc-tag are indicated. B. Pull-down using the GST-TSC1 fusion proteins. Upper panel: immunoblot of HA-TBC1D7 retained on the glutathione-GST-TSC1 fusion protein beads. Lower panel: coomassie staining of the purified GST-TSC1 proteins. C. The integrated intensities of the immunoblot signals for bound TBC1D7 were quantified relative to the GST-TSC1 939–992 fusion protein (939 – 992 = 1) in at least 3 independent experiments. TBC1D7 was retained significantly less effectively by GST fusion proteins lacking amino acids 939 – 992 (GST-RHO or delex22 879-myc) (unpaired t-test p values<0.05; indicated with asterisks). Error bars indicate the standard error of the mean. D. Conservation of residues corresponding to *TSC1* exon 22. The human (ex22), chimpanzee (chimp), mouse, chicken (chick), pufferfish (fugu), fruit fly (gigas) and fission yeast (pombe) sequences are shown. Identical residues are highlighted. Amino acids tested for their involvement in binding TBC1D7 are given (var). Heptad repeats 25 – 29 are indicated in red (coil). E. Predicted effects on the TSC1 coiled coil region according to the COILS server. Coils probability scores calculated for a 28 residue window (default conditions) are shown for each variant (colour), compared to the wild-type sequence (TSC1; black). Heptads 25 – 29 are indicated in red. F. Pull-down using GST-TSC1 exon 22 fusion proteins. Binding of TBC1D7 to wild-type GST-TSC1 939–992 (TSC1) was compared to GST-RHO and 11 different TSC1 variants (E945A, Y948N, K952I, R953G, R953W, I954K, KI-IK (K952I-I954K double variant), L963S, Y966X, K977X and L978V). Upper panel: immunoblot of HA-TBC1D7 retained on the glutathione-GST fusion protein beads. Lower panel: immunoblot of the purified GST-TSC1 fusion proteins. G. The integrated intensities of the immunoblot signals for bound TBC1D7 were quantified relative to wild-type GST-TSC1 939–992 (TSC1 = 1) in at least 3 independent experiments. TBC1D7 was retained significantly less effectively by the E945A, Y948N, K952I, R953G, R953W, I954K, KI-IK and Y966X variants (unpaired t-test p values<0.05; indicated with asterisks). Error bars indicate the standard error of the mean.

**Figure 5:**
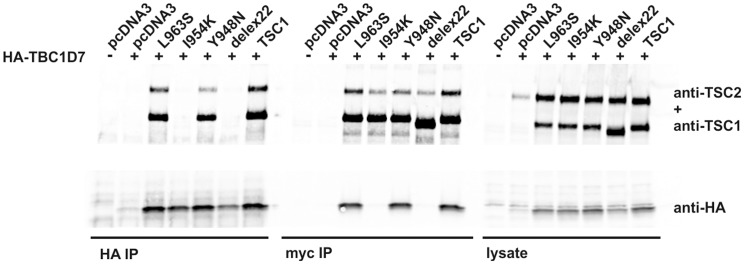
Functional assessment of the effect of the TSC1 Y948N, I954K and L963S variants on the TSC1-TSC2-TBC1D7 interaction. The TSC1 Y948N, I954K and L963S variants were coexpressed with TSC2 in transfected HEK 293T cells. TSC1-TSC2-TBC1D7 complexes were isolated by immunoprecipitation. Immunoblot showing expression of the TSC1 variants (anti-myc), TSC2 (anti-TSC2) and TBC1D7 (anti-HA) in the lysates of the transfected cells (right panels) and in anti-myc (myc IP; central panels) and anti-HA (HA-IP; left panels) immunoprecipitates. TBC1D7 was not detected in the TSC1-I954K or TSC1delex22 immunoprecipitates while TSC1-I954K and TSC1delex22 were not detected in the HA-TBC1D7 immunoprecipitates.

**Figure 6:**
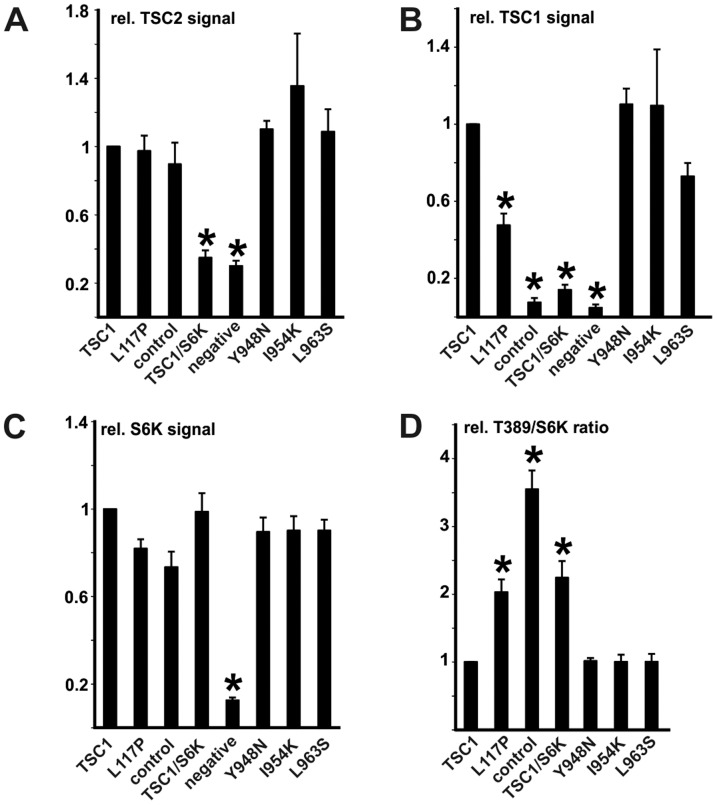
Assessment of the effects of the TSC1 Y948N, I954K and L963S variants on TORC1 signaling. The TSC1 variants were coexpressed in transfected HEK 293T cells with TSC2 and S6K. TSC2, TSC1, S6K and T389-phosphorylated S6K (T389) signals were estimated by immunoblotting. The TSC1 variants were compared to wild-type TSC1, the pathogenic TSC1 L117P variant, cells expressing TSC2 and S6K only (control), cells expressing TSC1 and S6K only and mock transfected cells (negative) in at least 3 independent experiments. The integrated intensities of the immunoblot signals for TSC2 (A) TSC1 (B) total S6K (C) and the T389/S6K ratio (D) were calculated relative to wild-type TSC1 (TSC1; = 1). Significant differences (unpaired t-test p values<0.05) are indicated with asterisks. Error bars indicate the standard error of the mean. No significant differences between wild-type TSC1 and the TSC1 Y948N, I954K or L963S variants were detected.

Next, we compared the binding of TBC1D7 to a series of bacterially expressed GST-TSC1 fusion proteins (Figure 4A). As shown in Figure 4B and C, TSC1 amino acids 939 – 992 (GST-TSC1ex22) were sufficient to bind TBC1D7. Fusion proteins lacking these residues, or containing an E945A substitution that was introduced during the cloning procedure, were much less effective at binding TBC1D7. E945 corresponds to the hydrophilic *e* residue of heptad 25 of the coiled coil domain (Figure 4D) and the E945A substitution is predicted to disrupt the structure of heptads 25 – 27 (Figure 3A and 4E). To investigate the TSC1-TBC1D7 interaction further, we derived a series of missense and truncated forms of the GST-TSC1 exon 22 fusion protein and compared their ability to bind TBC1D7 (Figure 4D, F and G). GST-TSC1ex22 Y966X, terminates after heptad 27; GST-TSC1ex22 K977X terminates at the *b* residue of heptad 28; GST-TSC1ex22 Y948N, corresponds to the hydrophobic *a* residue of heptad 25; GST-TSC1ex22 K952I, R953W, R953G and I954K correspond to the hydrophilic *e*, *f* and *g* positions of heptad 25; GST-TSC1ex22 L963S is in the linker region between heptads 27 and 28; and the GST-TSC1 L978V variant (see above) maps to the hydrophobic *d* position of heptad 28. The predicted effects of these changes on the propensity of TSC1 to assume a coiled coil conformation are summarised in Figure 4D. GST-TSC1ex22 K977X, L963S and L978V still bound TBC1D7, whereas GST-TSC1ex22 Y966X and the E945A, Y948N, K952I, R953G, R953W and I954K substitutions all significantly reduced TBC1D7 binding (Figure 4F and G). Furthermore, a fusion protein containing both the K952I and I954K substitutions (KI-IK) was unable to efficiently bind TBC1D7. To investigate whether the pull-down experiments reflected the behavior of full-length TSC1, we generated full-length TSC1 expression constructs containing the Y948N, I954K and L963S substitutions. Both the TSC1 Y948N and L963S variants retained the capacity to bind TBC1D7, whereas no interaction was detected between TBC1D7 and the TSC1 I954K variant (Figure 5). The I954K substitution had the same effect as complete removal of amino acids 938 – 991 (delex22). Interestingly, coimmunoprecipitation of TSC2 appeared slightly reduced in the presence of TSC1delex22 or the TSC1 I954K variant. Nonetheless, we did not observe significant effects of the TSC1 Y948N, I954K or L963S variants on the ability of the TSC1-TSC2 complex to inhibit TORC1 signaling in our standard assay (Figure 6).

### Amino acid substitutions mapping to TBC1D7 exons 4 and 5 prevent TSC1 binding

**Figure 7:**
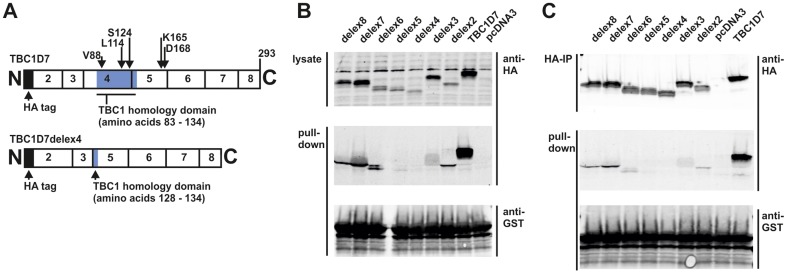
The TBC homology domain of TBC1D7 is required for the interaction with TSC1. Sequences corresponding to the individual coding exons of *TBC1D7* were deleted from the HA-tagged wild-type expression construct, maintaining the original reading frame (see Supporting Information Table S2 for details). TBC1D7delex proteins were expressed in transfected HEK 293T cells and interaction with TSC1 amino acids 939 – 992 was investigated by GST pull-down assay. GST TSC1 939 – 992 was expressed in *E. coli* and purified on glutathione-agarose beads. A. Schematic overview of TBC1D7 (above) and TBC1D7delex4 (below) showing the segments corresponding to each coding exon and the TBC-homology domain (shaded blue). Wild-type TBC1D7 and all the TBC1D7-derived delex proteins carried an N-terminal HA epitope tag, indicated as a filled (black) segment. The positions of amino acids tested for their involvement in TSC1 binding are indicated. B. Immunoblot analysis to show expression of the TBC1D7delex proteins in transfected HEK 293T cells (upper panel) and their retention on glutathione beads bound with GST-TSC1 939 – 992 (middle panel). The GST fusion protein used for the pull-down is shown in the lower panel. C. Immunoblot analysis to show recovery of the TBC1D7delex proteins by immunoprecipitation (upper panel) and their retention on glutathione beads bound with GST-TSC1 amino acids 939 – 992 (middle panel). The GST fusion protein used for the pull-down is shown in the lower panel.

**Figure 8:**
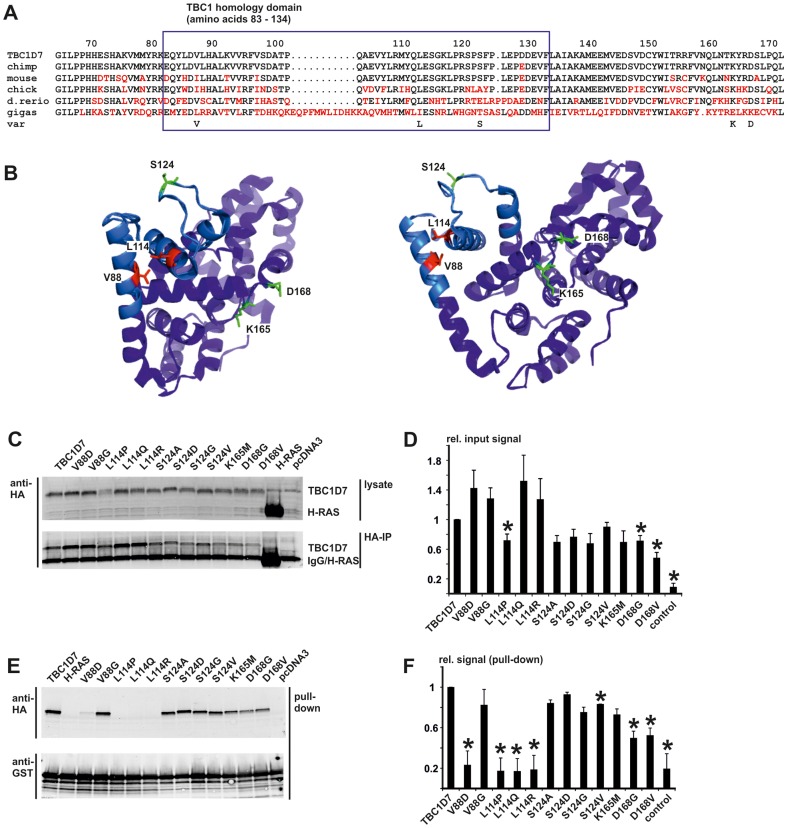
Effect of TBC1D7 amino acid substitutions on the interaction with TSC1. Interactions between TBC1D7 variants and the GST-TSC1 939–992 fusion protein were assessed by glutathione bead pull-down followed by immunoblot analysis. The TBC1D7 variants were expressed in transfected HEK 293T cells. GST TSC1 939 – 992 was expressed in *E. coli* and purified on glutathione-agarose beads. A. Detailed view of the evolutionary conservation of the TBC1D7 TBC homology domain. The human sequence (TBC1D7) was compared to chimpanzee (chimp), mouse, chicken (chick), zebrafish (d.rerio) and fruit fly (gigas). Non-identical residues are highlighted in red. Variant amino acids (var) tested for their effect on the binding with TSC1 are indicated below the alignment. B. Structural representation of TBC1D7 as determined by X-ray crystallography. The TBC homology domain is indicated by the light-blue shading. The positions of amino acids V88 and L114 are indicated in red; amino acids S124, K165 and D168 are shown in green. C. Immunoblot analysis of TBC1D7 variants. Expression (upper panel) and immunoprecipitation (lower panel) of the HA-tagged TBC1D7 variants was compared to the wild-type protein (TBC1D7), HA-tagged H-RAS (H-RAS) and mock-transfected cells (pcDNA3). D. The integrated intensities of the immunoblot signals of the expressed TBC1D7 variants were quantified relative to the wild-type protein (TBC1D7 = 1) in at least 3 independent experiments. Significant differences (paired t-test p values<0.05) are indicated with asterisks. Error bars indicate the standard error of the mean. E. Immunoblot analysis to show retention of the TBC1D7 variants (upper panel) by glutathione beads bound to the GST-TSC1 939–992 fusion protein (lower panel). F. The integrated intensities of the immunoblot signals of the TBC1D7 variants retained on the glutathione beads were quantified relative to the wild-type protein (TBC1D7 = 1) in at least 3 independent experiments. Significant differences (paired t-test p values<0.05) are indicated with asterisks. Error bars indicate the standard error of the mean.

**Figure 9:**
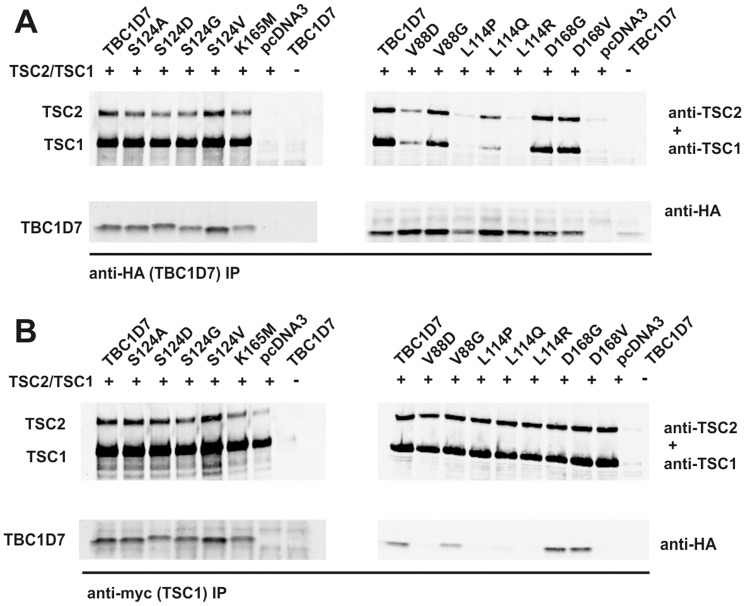
Effect of TBC1D7 amino acid substitutions on the TSC1-TSC2-TBC1D7 complex. To assess the effects of the TBC1D7 variants on the TSC1-TSC2-TBC1D7 interaction, the TBC1D7 variants were coexpressed with TSC1 and TSC2 in transfected HEK 293T cells. A. Immunoblots showing immunoprecipitation of the TBC1D7 variants (anti-HA IP) and coimmunoprecipitation of TSC1 and TSC2. B. Immunoblots showing coimmunoprecipitation of the TBC1D7 variants and TSC2 with TSC1 (anti-myc IP).

To try and identify *TBC1D7* variants that could affect the TBC1D7-TSC1 interaction we screened the coding exons of *TBC1D7* in 19 individuals with TSC in whom no *TSC1* or *TSC2* mutation had been identified. We detected 3 previously identified single nucleotide polymorphisms (SNPs) in *TBC1D7* but no pathogenic mutations in our cohort (Table 1), consistent with previous observations [18]. As an alternative approach, we compared the effects of in-frame deletion of the amino acids corresponding to each of the coding exons of *TBC1D7* (exons 2 – 8; referred to as TBC1D7delex2, TBC1D7delex3 *etc*; Figure 7A and Supporting lnformation Table S2) on the TSC1-TBC1D7 interaction. Deletion of exon 4 (TBC1D7delex4; amino acids 66 –127) or 5 (TBC1D7delex5; amino acids 128 – 173) disrupted the interaction with the GST-TSC1 exon 22 fusion protein (Figure 7B and C). Therefore, we investigated the effects of amino acid substitutions mapping to these exons on the TBC1D7-TSC1 interaction (Figure 8). V88 and L114 are two conserved residues in the TBC domain (amino acids 83 – 134) [19] and X-ray crystallography data indicates that these residues are located close together on adjacent alpha-helices [27](Figure 8B); according to Scansite (http://scansite3.mit.edu/#home), S124 is a potential basophilic kinase phosphorylation site; and K165 and D168 are outside the TBC domain but are conserved residues exposed on the surface of TBC1D7 (Figure 8B). All the TBC1D7 variant proteins were expressed and could be immunoprecipitated (Figure 8C and D). The L114P L114Q L114R and V88D substitutions clearly disrupted the interaction between TBC1D7 and the GST-TSC1 exon 22 fusion protein whereas the V88G, S124A, S1245D, S124G, D168G, D168V and K165M substitutions had less drastic effects (Figure 8E and F). To confirm the results of the pull-down experiments we coexpressed the TBC1D7 variants together with TSC1 and TSC2 in HEK 293T cells and isolated TBC1D7-TSC1-TSC2 complexes by immunoprecipitation of either the TBC1D7 or TSC1 subunit (Figure 9). The TBC1D7 V88D, L114P, L114Q and L114R variants were not detectable in the TSC1 immunoprecipitates, and coimmunoprecipitation of the TSC1-TSC2 complex with these variants was clearly reduced. In contrast, the TBC1D7 V88G, S124A, S124D, S124G, S124V, K165M, D168G and D168V substitutions did not affect TBC1D7-TSC1-TSC2 coimmunoprecipitation.

**Table 1:** Polymorphisms identified in *TBC1D7* in 28 individuals with TSC in whom no *TSC1* or *TSC2* mutation had been identified during routine mutation screening.

exon	cDNA	SNP	genomic position (build hg 19; chr 2)	heterozygote individuals
**3**	c.193+47G>A	rs2439553	g.13325279	5 (26%)
**4**	c.381+43C>T	rs2496132	g.13321097	1 (5%)
**6**	c.665+8G>A	rs2439537	g.13307824	4 (21%)

## Discussion

**Figure 10:**
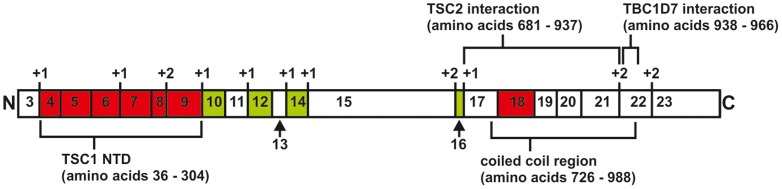
Overview of functional domain structure of TSC1. The segments encoded by each *TSC1* coding exon (3 – 23) are shown. Intron-exon boundaries that are not exactly in-frame are indicated with +1 or +2. Exons 4 – 9 and 18 that are essential for TSC1 stability are shown in red and the extent of the TSC1 coiled coil region and the regions required for the interactions with TSC2 and TBC1D7 are indicated. Exons that do not appear to be critical for TSC1 function are shaded green.

TSC1 is an essential component of the TSC1-TSC2-TBC1D7 complex. Apart from the predicted coiled coil domain in the C-terminal half, TSC1 does not show significant homology to other proteins and it has therefore been difficult to unravel the domain structure. To obtain additional insight into the structure and function of TSC1, we generated a series of exon-specific in-frame deletion mutant proteins. We assayed the effects of these deletions on the activity of the TSC1-TSC2 complex and on the ability of TSC1 to interact with TSC2 and TBC1D7. Our results are summarised in Figure 10.

Deletion of the amino acids encoded by exons 10, 12, 14 or 16 did not affect TSC1 function in our assays. If this would also be the case *in vivo*, it may be possible to rescue the TSC phenotype in individuals with mutations in exon 14 by designing antisense oligonucleotides to induce skipping of this exon, as has been described for other diseases [28]. Skipping of exons 10, 12 or 16 would result in a frameshift, so would not be amenable to this approach. Consistent with the apparent redundancy of the sequences encoded by these exons, functional assessment of 10 different amino acid changes mapping to these exons has so far failed to identify any effect on TSC1-TSC2 function [13], [14]. Deletion of the amino acids encoded by exon 9, 18 or 23 significantly reduced the levels of TSC1, most likely due to an effect on TSC1 stability. Previous work has highlighted the importance of the TSC1 NTD [11], [12], [25] for TSC1 stability. Our new data indicate that amino acids 247 – 304 are also important for the formation and/or stability of the NTD. Furthermore, our data suggest that amino acids 737 – 797 (exon 18), corresponding to heptads 2 – 6 of the coiled coil region, and amino acids 993 – 1164 are required for TSC1 stability. However, consistent with previous findings showing that the TSC1-TSC2 interaction is mediated through multiple regions of TSC1, TSC2 could still be coimmunoprecipitated with the TSC1 delex9, delex18 and delex23 proteins. Indeed, none of the exon-specific deletion proteins completely prevented TSC1-TSC2 binding (Figure 2; see below).

Exons 17 and 19 – 21 encode heptads 1–2 and heptads 7 – 23 respectively of the TSC1 coiled coil region. The absence of these sequences did not significantly affect TSC1 expression levels, but did reduce TSC2 coimmunoprecipitation, confirming the importance of the coiled coil region for the TSC1-TSC2 interaction. Given the above observations it was initially somewhat surprising that deletion of exon 22, encoding heptads 24 – 29 of the coiled coil region, did not significantly affect TSC1 immunoprecipitation or the TSC1-TSC2 interaction as assayed by coimmunoprecipitation of TSC2. However, our data indicate that this part of the coiled coil region is essential for the interaction with TBC1D7, consistent with previous observations [16]. Our data help further delineate the TBC1D7 binding domain of TSC1. Our results indicate that TBC1D7 binds specifically to heptads 24 – 27 of the TSC1 coiled coil region, providing an explanation for why these sequences are not directly involved in the interaction with TSC2.

To gain more insight into the TSC1-TBC1D7 interaction, we investigated the effects of different amino acid substitutions on the binding between TBC1D7 and the GST-TSC1ex22 fusion protein. We observed the greatest effect with the TSC1 Y948N, K952I and I954K substitutions, and the TBC1D7 V88D and L114P, L114Q and L114R substitutions. The TSC1 Y948N, K952I and I954K substitutions map to heptad 26 of the coiled coil region and are predicted to alter the helical structure of the last segment of this region (Figure 4E). However, other substitutions such as E945A, R953G and R953W, were also predicted to affect the coiled coil structure and had less drastic effects on the interaction with TBC1D7. We did not identify a clear correlation between the effects of the substitutions on the likelihood of the sequence to assume a coiled coil conformation and their effects on TBC1D7 binding. Therefore, it may not be the TSC1 coiled coil structure *per se* that is required for the TSC1-TBC1D7 interaction, but that specific residues within the coils are critical. Interestingly, when we introduced the Y948N substitution into the full-length TSC1 protein, the effect on TBC1D7 binding was less dramatic. It is possible that the surrounding sequences help maintain amino acids 939 – 977 in the correct conformation for TBC1D7 binding, and that the GST-TSC1ex22 fusion protein is more likely to undergo conformational change. This could be important for further *in vitro* analysis of the effects of amino acid changes on TSC1 function: *in vitro* produced TSC1, especially when consisting of just a portion of the protein, may be more sensitive to amino acid substitutions than full-length TSC1. TSC1, TSC2 and TBC1D7 homologs are expressed in *D. melanogaster* [18]. Interestingly, the TSC1 K952 and I954 residues that are essential for the TSC1-TBC1D7 interaction are both conserved in *D. melanogaster* (Figure 4D). This suggests that the TSC1-TSC2-TBC1D7 interaction may also be conserved. In summary, our data indicate that amino acid residues in heptad 26 of the TSC1 coiled coil region are essential for the binding with TBC1D7.

Previous work [17] had shown that a peptide corresponding to TBC1D7 amino acids 112 – 171 disrupted the TSC1-TBC1D7 interaction. We found that deletion of either amino acids 66 – 127 (TBC1D7delex4) or 128 – 173 (TBC1D7delex4) of TBC1D7 prevented TSC1 binding. Furthermore, we demonstrated that two conserved residues mapping to adjacent alpha helices, V88 and L114, were critical for the TSC1-TBC1D7 interaction.

In summary, we have employed an exon-deletion strategy to characterise distinct structural and functional regions in TSC1 and TBC1D7, two components of the "Rhebulator" (TSC1-TSC2-TBC1D7) complex [18]. TSC1 amino acids 247 – 304 (TSC1delex9) and 737 – 797 (TSC1delex18) are essential for TSC1 stability and function. In contrast, amino acids 306 – 343 (TSC1delex10), 382 – 421 (TSC1delex12), 445 – 479 (TSC1delex14) and 667 – 680 (TSC1delex16) have no effect on TSC1-TSC2 function in our *in vitro* assays. Although it is possible that alterations in these sequences would impair TSC1-TSC2 function *in vivo*, it is also possible that some residual TSC1-TSC2 activity would remain. Heptads 1 – 24 of the TSC1 coiled coil region are important for the interaction with TSC2, while heptads 25 – 27 are essential for the interaction with the TBC1 homology domain of TBC1D7. According to our analysis, specific changes to the conserved V88 and L114 residues encoded by *TBC1D7* exon 4 completely prevent the TSC1-TBC1D7 interaction. Our work gives more detailed insight into the structural and functional relationships between TSC1 and TBC1D7, and provides useful information for further studies aimed at unravelling the molecular structure of the TSC1-TSC2-TBC1D7 complex.

## Supporting Information

Table S1 **Overview of the TSC1delex expression constructs.**
(DOCX)

Table S2 **Overview of the TBC1D7delex expression constructs.**
(DOCX)
