# Supplementary material for: Identification of Regions Critical for the Integrity of the TSC1-TSC2-TBC1D7 Complex
Source: PLoS One. 2014 Apr 8;9(4):e93940. doi: 10.1371/journal.pone.0093940 (PMC3979717; doi:10.1371/journal.pone.0093940)
Supplement: Table S1 — Overview of the TSC1delex expression constructs. (DOCX) [file pone.0093940.s001.docx]

**Supporting Information Table S1.** Overview of the TSC1delex expression constructs. The nucleotide and amino acid sequences surrounding the deleted exon in the TSC1 cDNA are shown, with numbering according to Genbank accession number gi:2331280. The TSC1delex constructs are listed, with the corresponding deleted amino acid. Nucleotides corresponding to the site-directed mutagenesis primers are shown in bold. The first and last nucleotides of the deleted exon are underlined. In each case, deleted nucleotides and amino acids are shown in normal type. Nucleotides corresponding to the expression vector are in italics and amino acids from the C-terminal myc epitope tag linker sequence are indicated in underlined italics.

TSC1delex9 726 **ggaccctcgaagg**tgg...tat**gggtgtgctac**

(del W247 - Y304) 243 **D P R R** W Y **G C A**

TSC1delex10 901 **cagaatagctatgg**gtgt...caa**gctactctttggagc**

(del C306 - Q343) 301 **Q N S Y G** C Q **A T L W S**

TSC1delex12 1126 **gtctttggtacaactgca**ggt**...**aag**gaagagagaatggattctgc**

(del G382 - K421) 376 **V F G T T A** G K **E E K M D Y**

TSC1delex14 1317 **gaatgacagaggatca**gaa**...**gaa**gctgcaatatctagagaac**

(del E445 - E479) 440 **N D R G S** E E **A A I S R E**

TSC1delex16 1986 **ggagctgaacaag**ttg...gga**ggctctcctccttc**

(del L667 - G680) 663 **E L N K** L G **G S P P**

TSC1delex17 2027 **ggacccactttggaggc**tct...atg**aaagatcagttgaag**

(del S682 - M736) 677 **T H F G G** S M **K D Q L K**

TSC1delex18 2194 **cataatgctgccatg**aaa...cag**acgaagctggaggac**

(del K737 - Q797) 732 **H N A A M** K Q **T K L E D**

TSC1delex19 2373 **ccagagccaggaattacag**acg...aag**ctctcaaacagtgagtcg**

(del T798 - K834) 792 **Q S Q E L Q** T K **L S N S E S**

TSC1delex20 2486 **gtcaggtttcccaaaag**ctc...aag**gaagtagaaatgatgaaagcc**

(del L835 - K875) 830 **Q V S Q K** L K **E V E M M K A**

TSC1delex21 2612 **cagataccacaaag**gaa...aga**ggacagctgcaggcc**

(del E876 - R938) 872 **D T T K** E R **G Q L Q A**

TSC1delex22 2799 **caaactccaggca**aga...gaa**aggcttgactgttgtaatg**

(del R938 - E991) 934 **K L Q A** R E **R L D C C N**

TSC1delex23 2962 **gcagcagaagaaagg**ctt...agc***ggtaccaagcttggg***

(del L993 - S1164) 988 **A A E E R L S *G T K L G***
