# Supplementary material for: Identification of Regions Critical for the Integrity of the TSC1-TSC2-TBC1D7 Complex
Source: PLoS One. 2014 Apr 8;9(4):e93940. doi: 10.1371/journal.pone.0093940 (PMC3979717; doi:10.1371/journal.pone.0093940)
Supplement: Table S2 — Overview of the TBC1D7delex expression constructs. (DOCX) [file pone.0093940.s002.docx]

**Supporting Information Table 2.** Overview of the TBC1D7delex expression constructs. The nucleotide and amino acid sequences surrounding the deleted exon in the TBC1D7 cDNA are shown, with numbering according to Genbank accession number gi:301500687. The TBC1D7delex constructs are listed, with the corresponding deleted amino acids. Nucleotides corresponding to the site-directed mutagenesis primers are shown in bold. The first and last nucleotides of the deleted exon are underlined. In each case, deleted nucleotides and amino acids are shown in normal type. Nucleotides corresponding to the expression vector are in italics and amino acids from the N-terminal HA epitope tag linker sequence are indicated in underlined italics.

TBC1D7delex2 1 ***gctagcctcggtgaattc***atg...ctg**gatactgagaaactttg**

(del M1 - L37) 1 ***A S L G E F*** M L **D T E K L**

TBC1D7delex3 100 **gatgaccgtctggat**act...gga**atcttgcctccacacc**

(del T39 - G65) 34 **D D R L D** T G **I L P P H**

TBC1D7delex4 179 **ggaaggtgcttctagga**atc**...**ctg**gagccagatgatgaag**

(del I66 - L127) 61 **K V L L G** I L **E P D D E**

TBC1D7delex5 367 **ccctcttttccactg**gag...ttg**ccaaaagcgtttgaac**

(del E128 - L173) 123 **P S F P L** E L **P K A F E**

TBC1D7delex6 506 **ccttgccccagttg**cca...agg**gtttgggataaagttg**

(del P174 - R222) 170 **L P Q L** P R **V W D K V**

TBC1D7delex7 653 **ccagtttacagagg**gtt...aat**attccccaggacagc**

(del V223 - N265) 219 **S L Q R** V N **I P Q D S**

TBC1D7delex8 779 **caaagtttctggaaaat**att...aac**tga*ctcgagcatgcatc***

(del I266 - N319) 261 **K F L E N** I N *****
